# Hepatocellular carcinoma muscle and skin metastasis: A case-report

**DOI:** 10.1016/j.radcr.2026.05.067

**Published:** 2026-06-17

**Authors:** Mahyar Daskareh, Reihaneh Mortazavi Ardestani, Ali Nosrati Andevari, Fatemeh Abbasi

**Affiliations:** a Department of Radiology, Ziaeian Hospital, Tehran University of Medical Sciences, Tehran, Iran; b Department of Radiology, Hospital of the University of Pennsylvania, Philadelphia, PA, USA; c Advanced diagnostic and interventional radiology research center (ADIR), Tehran University of medical sciences, Tehran, Iran; d Department of Clinical Biochemistry, Afzalipour Faculty of Medicine, Kerman University of Medical Sciences, Kerman, Iran; e Department of Medicine, To.C., Islamic Azad University, Tonekabon, Iran; f Clinical Research Development Unit of Akhtar Hospital, Shahid Beheshti University of Medical Sciences, Tehran, Iran

**Keywords:** Hepatocellular carcinoma, Skeletal metastasis, Magnetic Resonance Imaging, Soft tissue metastasis

## Abstract

A 55-year-old patient with chronic hepatitis B infection who developed hepatocellular carcinoma. The tumor was managed with primary therapeutic resection. On postoperative follow-up imaging, rising alpha-fetoprotein noted which led into contrast-enhanced MRI of abdomen. On Imaging review, an abdominal wall lesion suspected, which was more conspicuous on the post contrast images. There are several differential diagnosis for these soft tissue lesions which demonstrate many shared imaging characteristics. Musculoskeletal and cutaneous metastases from hepatocellular carcinoma are uncommon, though clinically critical since it changes management. We strongly recommend detailed evaluation of skin and musculoskeletal regions among these patients.

## Introduction

Hepatocellular carcinoma (HCC) ranks as the sixth most prevalent primary malignancy and is the fourth leading cause of cancer-related mortality worldwide [1]. This primary hepatocellular neoplasm is highly aggressive, and metastasizes both intra-hepatically and extrahepatically, with extrahepatic metastasis occurring in approximately 15%-17% of patients, more common in the lungs, regional lymph nodes, bones, adrenal glands, and peritoneum [2]. It is relatively rare for HCC tumors to metastasize to the musculoskeletal (MSK) structures via hematogenous spread. MSK metastases are less common, likely because of constant movement and tension, which make these areas less conducive for tumor growth [3]. These metastases are typically identified through needle biopsy or during follow-up imaging. While needle biopsies are useful for diagnosing such metastases, they pose the risk of tumor cell dissemination along the biopsy track [2,4]. Moreover, muscle tissues contain proteases and various inhibitors that may inhibit the invasive growth of tumors [5].

Skeletal and skin HCC metastases would present with clinical symptoms such as pain, neurologic symptoms, subcutaneous swelling and mass, and pathologic fracture [6]. In addition, elevated AFP in patients with treated HCC without evidence of local recurrence may serve as a diagnostic clue when a radiologist discover a new soft tissue abnormality on imaging [7]. Furthermore, this tumor biomarker correlate well with metastasis size and its aggressiveness [8].

MSK metastases is often an incidental finding on imaging during baseline staging or response to treatment assessment. The presence of skeletal muscle metastasis typically signals aggressive tumor behavior, often representing the terminal stages of the disease [9,10] and contrast-enhanced Computed Tomography (CE-CT) is a suitable imaging modality and technique [11]. In comparison to CE-CT, Magnetic Resonance Imaging (MRI) would help us to differentiate different muscular lesions such as HCC metastasis in oppose to primary benign lesions [12]. This underscores the need for radiologists to promptly detect such extrahepatic metastases and accurately stage the disease [2,13].

In this study, we shed light on the importance of radiologist's responsibility in searching for these metastases among HCC patients on screeening and follow-up imaging, which can consequently change the treatment plan.

## Case presentation

A 55-year-old man with chronic hepatitis B virus (HBV) infection following up in hepatology clinic. No past medical history of chronic disease apart from HBV infection. No noticeable family history of chronic hepatic disease or malignancy. On routine clinical and laboratory assessment of chronic HBV infection, rising serum AFP of 2350 ng/mL (adult nonpregnant normal range: 0 to 10 ng/mL) detected, eventhough no clinical symptom or sign of acute disease mentioned. Subsequent abdominal imaging revealed a solid hypervascular hepatic lesion. Thorough imaging assessment and staging proved potential malignant lesion without metastasis. As a result, primary tumor resection was done as the best next step for management. No neoadjuvant or adjuvant systemic treatment or radiation were done. Postoperative recovery was uncomplicated, and patient had a substantial biochemical response, with AFP decreasing to 8 ng/mL 3 months after surgery.

At 2-year surveillance visit, patient denied abdominal pain, any palpable mass, or other systemic complaints. Examination revealed a well-healed left subcostal scar, without abdominal-wall tenderness, swelling, or palpable nodules. There was no jaundice, ascites, or hepatomegaly. Routine laboratory evaluation revealed stable liver function test; alanine aminotransferase (ALT) 68 U/L (normal range: 10-49 U/L), aspartate aminotransferase (AST) 52 U/L (normal range: 0-35 U/L), alkaline phosphatase (ALP) 145 U/L (normal range: 44-121 U/L), and total bilirubin 1.1 mg/dL (normal range: 0.3-1.2 mg/dL). However, AFP had increased mildly to 62 ng/mL (normal range: 0-10 ng/mL) compared with the previous value of 8 ng/mL. Given the rising AFP trend despite the absence of symptoms, a surveillance MRI of the abdomen was performed.

Abdominal MRI revealed an intact surgical margin in the liver without suspicious findings of local recurrence or lymphadenopathy; however, an enhancing soft-tissue nodule in the anterior abdominal wall at the site of anterior abdominal incision. On axial T2-weighted imaging (T2WI), this lesion was isointense relative to the rectus abdominis muscle (Fig. 1A). On arterial-phase contrast-enhanced (CE) T1-weighted MRI (T1WI), it demonstrated diffuse non-rim enhancement (Fig. 1B), and on delayed-phase CE T1WI, it remained isointense (Fig. 1C). Findings were suspicious for potential new abdominal wall musculature metastasis.

**Fig. 1:**
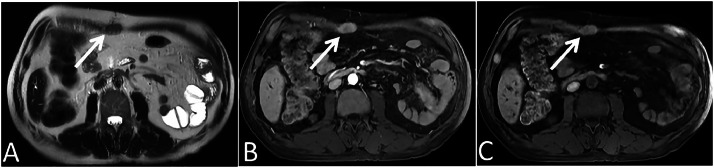
(A) Axial T2WI shows the lesion (arrows) to be isointense relative to the rectus muscle. (B) Axial CE T1WI in the arterial phase demonstrates diffuse non-rim enhancement of the lesion. (C) On delayed-phase CE T1WI the lesion remains isointense relative to the muscle. This case underscores the critical role of contrast-enhanced MRI in detecting subtle soft tissue metastases.

The patient was subsequently evaluated by a multidisciplinary tumor board to design best treatment planning for this newly diagnosed metastasis in the musculature. On the basis of presumed metastasis from HCC primary, local cytoablative therapy was considered with single agent Pembrolizumab for a course of 2 years. Following 3 sessions of cryoablations, targeted soft tissue ultrasound and MRI demonstrated a treatment cavity with internal hemorrhagic content. No enhancing component was identified in the ablation bed to suggest residual viable tumor. Additionally, serum AFP level was discovered to be within normal range on 18 months follow-ups.

We also added a companion case with appreciable MRI findings to provide more examples. Fig. 2 represents another patient with similar soft tissue metastases. A 60-year-old gentleman with history of pathology-proven HCC with pulmonary and nodal metastases, currently receiving multiagent systemic therapy. On physical examination, no physical symptoms or signs of new palpable abdominal wall lesion were noted.

**Fig. 2:**
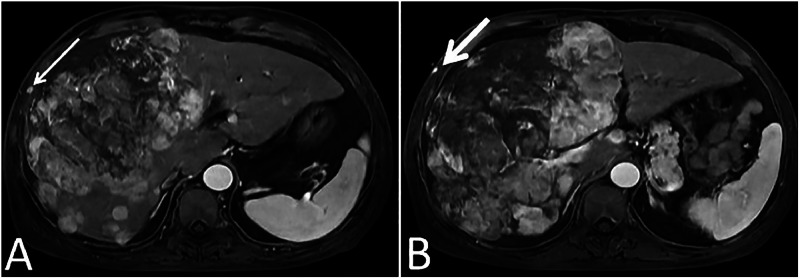
Companion case. A 60-year-old man with metastatic HCC. (A) Axial CE T1WI reveals a subtle implant within the anterolateral abdominal wall (arrow). (B) On an adjacent slice, a cutaneous metastatic implant (arrow) is denoted in the right anterior abdominal wall, involving skin, subcutaneous fat and likely musculature.

## Discussion

HCC metastases to the muscular and skin is rare and may present with imaging characteristics similar to the primary hepatic tumor [14]. In align with this concept, some case studies reports described MSK metastases with washout on the portal or equilibrium phases of dynamic imaging, which is a characetristic observation of HCC [2,15]. Of note, unenhanced CT or MRI might be less specific in providing such diagnosis. For isntance, Goto et al. described a HCC soft tissue metastasis with heterogenous T2 hypointensity and T1 mild hyperintensity, which are nonspecific MRI fearures [16].

Therefore, many pathologies can mimic HCC metastases to the muscles and skin, including desmoid, lymphoma, fibrosarcoma, surgical scar, endometriosis, sebaceous cyst, and myxoma. First differential diagnosis, desmoid tumors may appear heterogenous due to hemorrhage, necrosis, or calcification and iso-dense to muscle attenuation on CE-CT scan. Furthermore, desmoid MRI signal varies from high to low on T2WI as fibrous content increases, often demonstrating hypointense bands corresponding to collagen dense bundles, and usually enhances following gadolinium administration [4]. Next differential diagnosis is abdominal wall endometriosis, which is iso- to hyperintense relative to the muscle on T1WI on the basis of the methemoglobin content, and often enhances on CE-MRI [17]. Third differential diagnosis, sebaceous cysts, a type of epidermal lesion, generally appear as well-defined fluid-filled structures with low signal on T1WI and high signal on T2WI, and rim post contrast enhancement [5,9].

Fourth differential diagnosis of muscle and skin HCC metastases is myxoma, characterized by homogeneous low T1 and high T2 signal intensity on MRI [10]. Fifth differential is a less common fibrosarcoma which show iso-attenuated soft tissue masses on CT, low T1 signals, and mixed intensity on T2WI [11,12].

Beyond imaging differential diagnosis, physical examination findings such as inflamed skin, or sever local pain might lead us to a more benign acute inflammatory diagnosis or menstrual-related symptoms might help us to make the diagnosis of the endometriosis [18].

In terms of treatment strategies while treatment plans for primary HCC have evolved, standardized protocols for extrahepatic metastases remain lacking [19]. Surgical resection, particularly in cases of oligometastatic disease, remains the only potentially curative treatment and has been shown to improve survival in selected patients [20]. Percutaneous cryoablation introduced recently for HCC metastases. A recent study reported 2 cases of abdominal wall metastases treated using cryoablation technique with both patients experienced uneventful recoveries and demonstrated no radiographic evidence of recurrence at 12 and 18 months of follow-up [21].

The current short study emphasize the importance of muscle and skin investigation during HCC surveillance imaging. Radiologists are encouraged to adopt a structured checklist approach that specifically incorporates assessment of potential implantation sites, including prior biopsy tracts, surgical incisions, or procedural access points—ranging from chest wall thoracotomy incisions for pulmonary metastatectomy to ventral abdominal wall or percutaneous intervention sites. Even in asymptomatic patients, a rise in serum AFP should prompt thorough assessment.

## Conclusion

Musculoskeletal and cutaneous HCC metastases are uncommon and often clinically silent; however, it is clinically relevant, and best detected on contrast enhanced CT and MRI. These malignant implants need special attention to be picked up on surveillance imaging. In oligometastatic scenarios, local control with image-guided ablation can be considered.

## Patient consent

Written informed consent was obtained from the patient for publication and any accompanying images. A copy of the written consent is available for review by the Editor-in-Chief of this journal on request.
